# Transcription start site scanning requires the fungi-specific hydrophobic loop of Tfb3

**DOI:** 10.1093/nar/gkae805

**Published:** 2024-09-17

**Authors:** Chun Yang, Pratik Basnet, Samah Sharmin, Hui Shen, Craig D Kaplan, Kenji Murakami

**Affiliations:** Department of Biochemistry and Biophysics, Perelman School of Medicine, University of Pennsylvania, 415 CurieBlvd. Philadelphia, PA 19104, USA; Department of Biological Sciences, University of Pittsburgh, 5th and Ruskin Avenues, Pittsburgh, PA 15260, USA; Department of Biochemistry and Biophysics, Perelman School of Medicine, University of Pennsylvania, 415 CurieBlvd. Philadelphia, PA 19104, USA; School of Life Science and Technology, China Pharmaceutical University, 639 Longmian Road, Nanjing 210009, China; Department of Biological Sciences, University of Pittsburgh, 5th and Ruskin Avenues, Pittsburgh, PA 15260, USA; Department of Biochemistry and Biophysics, Perelman School of Medicine, University of Pennsylvania, 415 CurieBlvd. Philadelphia, PA 19104, USA

## Abstract

RNA polymerase II (pol II) initiates transcription from transcription start sites (TSSs) located ∼30–35 bp downstream of the TATA box in metazoans, whereas in the yeast *Saccharomyces cerevisiae*, pol II scans further downstream TSSs located ∼40–120 bp downstream of the TATA box. Previously, we found that removal of the kinase module TFIIK (Kin28–Ccl1–Tfb3) from TFIIH shifts the TSS in a yeast *in vitro* system upstream to the location observed in metazoans and that addition of recombinant Tfb3 back to TFIIH-ΔTFIIK restores the downstream TSS usage. Here, we report that this biochemical activity of yeast TFIIK in TSS scanning is attributable to the Tfb3 RING domain at the interface with pol II in the pre-initiation complex (PIC): especially, swapping Tfb3 Pro51—a residue conserved among all fungi—with Ala or Ser as in MAT1, the metazoan homolog of Tfb3, confers an upstream TSS shift *in vitro* in a similar manner to the removal of TFIIK. Yeast genetic analysis suggests that both Pro51 and Arg64 of Tfb3 are required to maintain the stability of the Tfb3–pol II interface in the PIC. Cryo-electron microscopy analysis of a yeast PIC lacking TFIIK reveals considerable variability in the orientation of TFIIH, which impairs TSS scanning after promoter opening.

## Graphical Abstract

**Graphical Abstract:**
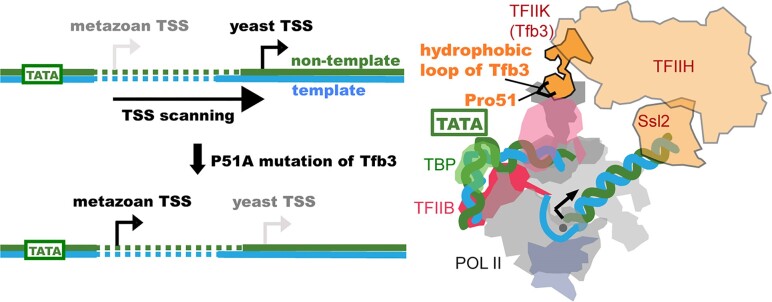

## Introduction

In eukaryotes, RNA polymerase II (pol II) transcription starts with the assembly of pre-initiation complex (PIC) at a TATA box or a TATA-like region on promoters (1). The PIC comprises the basal transcription machinery, which contains six general transcription factors (GTFs; TFIIA, TFIIB, TFIID or TBP, TFIIE, TFIIF, TFIIH) and pol II (2,3). Structural studies using cryo-electron microscopy (cryo-EM) revealed that PICs are highly conserved in eukaryotes with respect to overall conformation and components (4–9). However, PICs facilitate distinct transcription start site (TSS) utilization among species through highly conserved PIC components (10,11) and promoter sequences (12–14): in metazoans, transcription at TATA-containing promoters typically initiates ∼30–35 bp downstream of the TATA box (15), while in *Saccharomyces cerevisiae*, transcription initiates ∼40–120 bp downstream of the TATA box. Promoter melting begins ∼21 bp downstream in all eukaryotes (15), so pol II is presumed to scan further downstream before starting transcription in yeast (10,12,14,16–19). Real-time observations of single yeast PICs have demonstrated that the ATP-dependent translocase activity of Ssl2, the yeast homolog of XPB, continuously draws downstream DNA into the polymerase cleft, hypothesized to be in a partially unwound form, filling the single-stranded DNA-binding region of the pol II active center for TSS scanning (18). In the fission yeast *Schizosaccharomyces pombe*, transcription predominantly initiates ∼31 bp downstream of the TATA box as in metazoans, and to a lesser extent at multiple downstream TSSs within a window ranging from 30 to 70 bp downstream of the TATA box (20,21).

**Figure 1.**
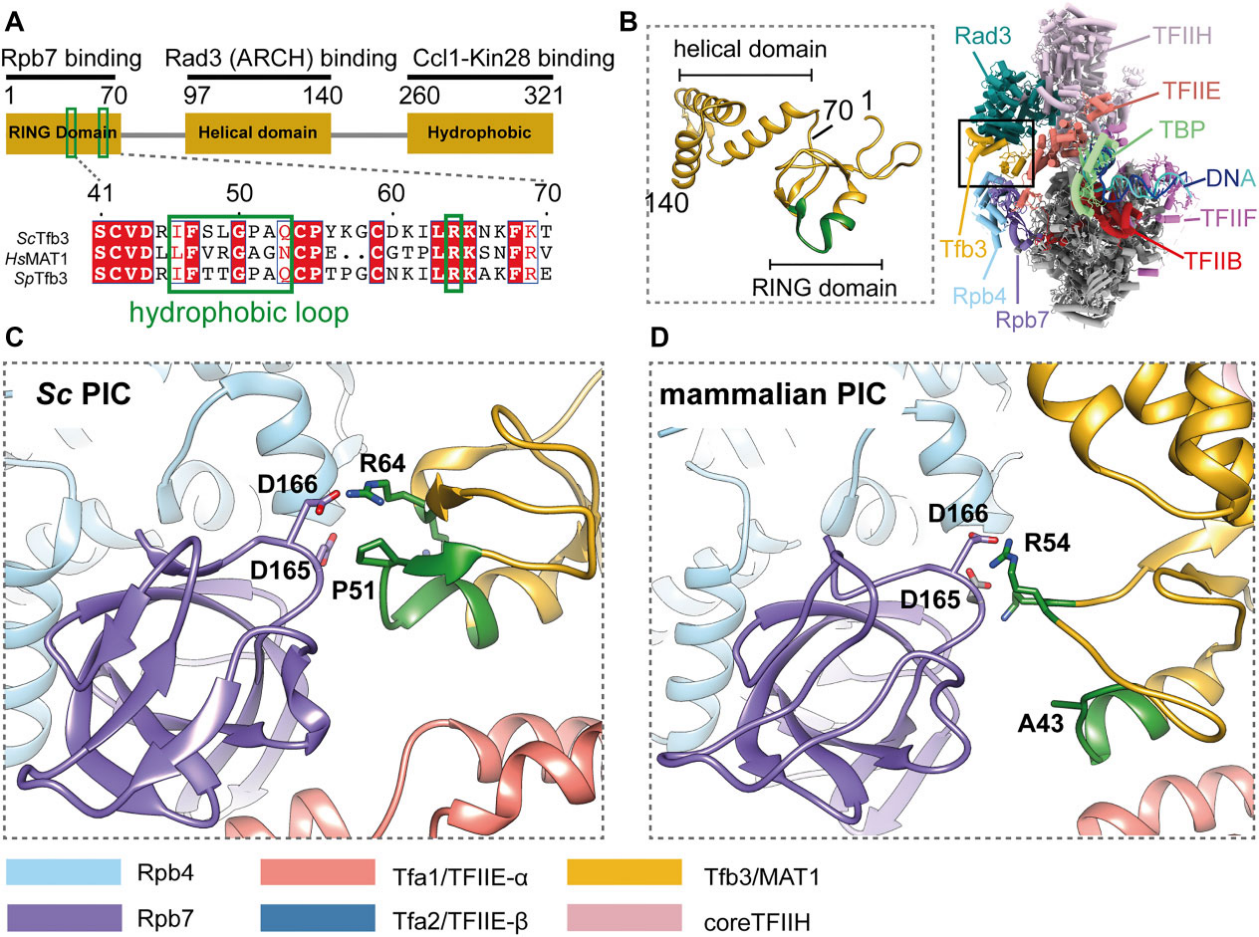
Structure of Tfb3/MAT1 in PICs. (**A**) Domain architecture of Tfb3. Hydrophobic loop and Arg64 are indicated by green boxes, with sequence alignment between *S. cerevisiae* (*Sc*), *Homo sapiens* (*Hs*) and *S. pombe* (*Sp*). (**B**) Structure of the N-terminal 148 residues of Tfb3 in the yeast PIC (PDB: 7ML0), bridging between TFIIH (Rad3 subunit in sea green) and pol II (Rpb4/7 subunits in sky blue/purple). (**C**) Interface between the RING domain of Tfb3 and Rpb7 in *Sc*PIC (PDB: 7o72). The hydrophobic loop and Arg64 are colored in green. (**D**) Interface between the RING domain of MAT1 and Rpb7 in the mammalian PIC (PDB: 7nvy).

In *S. cerevisiae*, TFIIH comprises a seven-subunit core complex, including the helicase Ssl2 (the yeast homolog of XPB), and a three-subunit kinase termed TFIIK. TFIIK, the yeast homolog of metazoan CAK, contains subunits Kin28, Ccl1 and Tfb3. Kin28 and Ccl1 (CDK7 and cyclin H in metazoans) constitute a cyclin-dependent kinase, responsible for pol II CTD phosphorylation during transcription initiation, while Tfb3, the yeast homolog of metazoan MAT1, serves to tether the cyclin kinase to the rest of the PIC by contacting pol II Rpb7, the ARCH domain of Rad3 (XPD of metazoan TFIIH), and Kin28/Ccl1 through the N-terminal RING domain (residues 1–70), the central α-helical domain (residues 97–140) and the C-terminal hydrophobic region (residues 260–321), respectively (Figure 1A) (22–24). Furthermore, TFIIK plays an important role in TSS scanning. In an *in vitro* reconstituted system from purified factors, omission of TFIIK shifts distal TSSs in a yeast system to a metazoan-like location (25), and addition of the N-terminal half of Tfb3 (residues 1–148) is sufficient to restore the TSS scanning ability (26). Consistent with this, when Tfb3 is split into N- and C-regions, yeast cells are viable, although the untethered kinase lost specificity for phosphorylating the pol II CTD at promoters and did so throughout transcribed genes (27).

Previous biochemical analysis suggested that electrostatic interaction between Rpb7 Asp166 and Tfb3 Arg64 (Figure 1A–C) is required for the activity of TFIIK in TSS scanning *in vitro* (26), consistent with a defect in TSS scanning *in vivo* for *Tfb3* alleles R64D and R64A and Rpb7 allele D166G. However, this electrostatic interaction between Rpb7 and Tfb3 is highly conserved across eukaryotes (Figure 1C and D, and Supplementary Figure S1), and thus is unlikely to explain the difference in TSS utilization between yeast and metazoans. In this study, we focused on another point of contact between Rpb7 and Tfb3, formed only in yeast PICs, but absent in metazoan PICs (Figure 1C and D). At this hydrophobic interface, Pro51, highly conserved only in fungi, within a loop region of Tfb3 (residues 46–53, referred to as the hydrophobic loop hereafter) is wedged between long aliphatic side chains of Lys164 and Glu165 of Rbp7. Disrupting this hydrophobic interaction by mutating Pro51 of Tfb3 to alanine or serine, as found in metazoans, virtually abolished TSS scanning *in vitro*. Yeast genetics experiments indicate that the hydrophobic and electrostatic interactions involving Pro51 and Arg64 at the Tfb3–pol II interface both contribute together to promote downstream TSS utilization. These findings, together with structural data, extend our current model of TSS scanning in yeast, with implications for how TFIIH works in other eukaryotes.

## Materials and methods

### Protein purification

TFIIA, TFIIB, TBP and Sub1 were purified recombinantly from bacteria. TFIIE, TFIIF, core TFIIH and pol II were purified from yeast as previously described (25). The Tfb3ΔC mutants were constructed by QuikChange site-directed mutagenesis on Tfb3ΔC (residues 1–148) as a template and purified as previously described (26).

### *In vitro* transcription initiation assay

*SNR20* 98W and *SNR20* 38D promoter templates were amplified by polymerase chain reaction and purified using Superose 6 10/300 (GE Healthcare) in buffer 300 [20 mM HEPES, pH 7.6, 300 mM potassium acetate, 5 mM dithiothreitol (DTT) and 2 mM magnesium acetate] (4). 1.3 pmol of PIC was formed on 1.3 pmol of DNA fragment with 2.0 pmol of TFIIA, 6.0 pmol of TFIIB, 12.0 pmol of TBP, 3.0 pmol of TFIIE, 5.2 pmol of TFIIF, 2.0 pmol of core TFIIH, 2.0 pmol of Sub1, 5.2 pmol of pol II and indicated amount of Tfb3 ΔC mutants in 5.0 μl of buffer 300 (50 mM HEPES, pH 7.6, 300 mM potassium acetate, 5 mM DTT and 5% glycerol). The mixture was diluted with 5.0 μl of buffer 10 (20 mM HEPES, pH 7.6, 10 mM potassium acetate, 5 mM magnesium sulfate and 5 mM DTT) and incubated on ice overnight. After 20 min of pre-incubation at 30°C, the transcription assays were initiated by addition of 10.0 μl of 2× NTPs containing 1.6 mM ATP, 1.6 mM CTP, 1 mM UTP, 1.6 mM GTP, 4.0 nM [α-^32^P] UTP (14.5 μCi), 1 unit of RNaseOUT and 10 mM magnesium acetate in buffer 10. The reaction was incubated for 20 min and stopped by addition of 190 μl of stop buffer [300 mM sodium acetate, pH 5.5, 5 mM ethylenediaminetetraacetic acid (EDTA), 0.7% sodium dodecyl sulfate (SDS), 0.1 mg/ml glycogen and 0.013 mg/ml of Proteinase K]. RNAs were recovered by ethanol precipitation and dissolved in formamide and subjected to a 6% urea acrylamide gel.

### Primer extension

Primer extension assay was performed as previously described (10) with modification. Briefly, RNAs were generated from the transcription assay *in vitro* as described earlier. Twenty microliters of transcription assays were stopped by addition of 190 μl of stop buffer without salmon sperm DNA (300 mM sodium acetate, pH 5.5, 5 mM EDTA, 0.7% SDS, 0.1 mg/ml glycogen and 0.013 mg/ml of Proteinase K). RNAs were recovered by ethanol precipitation, dissolved with RNase free water and then subjected to DNase I digestion to remove contamination of DNA template. Primers (Primer1: GAGGTCATTTCAGTTGTTACACTG; Primer2: AAGGGAAAAGGAAAAGAGATTTGT) were end-labeled with [γ-^32^P] ATP and annealed to RNAs. Reverse transcription was performed by adding M-MLV reverse transcriptase (Thermo Fisher) and RNase inhibitor (Thermo Fisher). RNase A was added to remove RNA after reverse transcription. Sequencing ladder was generated with a Sequenase Quick-Denature Plasmid Sequencing Kit with alkaline denaturation (USB). Products were analyzed by running a 7 M urea gel containing 6% acrylamide and visualized by phosphorimaging (Bio-Rad).

### Transcription-related growth phenotypes of *tfb3* alleles

Transcription-related growth phenotypes were analyzed as previously published (10,28). Yeast extract (1%, w/v; BD), peptone (2%, w/v; BD) and 2% bacto-agar (BD), supplemented with adenine (0.15 mM) and tryptophan (0.4 mM; Sigma–Aldrich), comprised YP solid medium. YPD plates contained dextrose (2%, w/v; VWR). Minimal media plates were prepared with synthetic complete (SC) or ‘Hopkins mix’ with appropriate amino acid(s) dropped out, with slight modifications as described in (29). Stock solutions (10 mg/ml, in 100% ethanol) of mycophenolic acid (MPA; Sigma–Aldrich) were added to solid or liquid media to achieve desired concentration. *Tfb3* alleles were generated by site-directed mutagenesis and verified by Sanger sequencing.

### Cryo-EM sample preparation and data collection of the PIC-ΔTFIIK

The PIC was prepared as follows: 0.6 nmol of a *HIS4* promoter DNA fragment was mixed with 1.0 nmol of TFIIB, 0.9 nmol of TFIIA, 0.7 nmol of TBP, 1.0 nmol of TFIIE, 0.4 nmol of TFIIH-ΔTFIIK, 0.66 nmol of pol II, 0.66 nmol of TFIIF and 1.2 nmol of TFIIS in 130 μl of buffer (500) (20 mM HEPES, pH 7.6, 5 mM DTT, 2 mM Mg(OAc)_2_ and 5% glycerol, with the millimolar concentration of KOAc in parentheses). The mixture was dialyzed into buffer (300), buffer (220), buffer (150), buffer (90) and buffer (40), and then was loaded onto a 10–40% (v/v) glycerol gradient containing 20 mM HEPES (pH 7.6), 5 mM DTT, 2 mM Mg(OAc)_2_ and 40 mM KOAc, and was centrifuged for 8 h at 36 000 rpm in a Beckman SW60 Ti rotor. For cryo-EM, PIC-ΔTFIIK was fixed by sedimentation in glycerol gradients containing a gradient of glutaraldehyde from 0 to 0.125%. Aliquots of peak fractions (∼0.3 mg/ml) were flash frozen in liquid nitrogen and stored until use at −80°C.

Samples were applied to R2/1 300 mesh Quantifoil holey carbon grids (Electron Microscopy Sciences). All grids were glow discharged (easiGlow, Pelco) for 2 min before deposition of 2 μl of dialyzed sample, and subsequently blotted for 2 s using Whatman Grade 41 filter paper (Sigma–Aldrich) and flash frozen in liquid ethane with a Leica EM CPC manual plunger (Leica Microsystems). Images were collected at Frederick National Laboratory (sponsored by the National Cancer Institute) using a NCEF Titan Krios transmission electron microscope operating at 300 kV, equipped with a K3 Bioquantum detector and a Bioquantum energy quantum filter. Three datasets were collected at a nominal magnification of 81 000× in super-resolution mode (pixel size of 0.54 Å) at a defocus range between 1 and 2.5 μm. A total of 23 569 images were collected. The exposure time was 3.2 s at a nominal dose of 50 e^−^/Å^2^. Movies were divided into 40 frames.

### Image processing and 3D reconstruction of the PIC-ΔTFIIK

The datasets were motion corrected with MotionCorr2 (30), and then CTF was estimated with CTFFIND4 (31). A total of 909 275 particles were extracted after particle picking using Topaz from the first dataset, and then the resultant particles were screened by three rounds of reference-free 2D classification, resulting in a subset of 593 614 particles showing PIC-like features. Subsequently, one round of 3D classification was carried out using a previous PIC map low-pass filtered to 60 Å resolution as a reference (26), yielding two reasonable 3D classes followed by one more round of 3D classification. Similar processes were applied to the second and third datasets. Briefly, particles were extracted on the coordination yielded with Topaz and then subjected to three rounds of reference-free 2D classification followed by two rounds of 3D classification. A total 780 331 particles from three datasets were combined and subjected to one round of 3D classification, yielding 744 986 good particles for 3D auto-refinement. A soft mask was created including TFIIH and TFIIE for signal subtraction, and then the subtracted images were used to generate an initial model. Then, 3D classification was performed with image alignment. The resulting classes revealed two distinct orientations of TFIIH relative to the rest of the PIC, accounting for 138 691 and 90 136 particles. To improve the map quality of TFIIH, particles were re-extracted without binning, followed by 3D auto-refinement without and with soft-edged mask for entire complex, followed by CTF refinement and Bayesian polishing, resulting in entire reconstruction of 3.7 and 3.8 Å resolution for forms 1 and 2, respectively. To further improve map quality of each form of PIC-ΔTFIIK, the maps were segmented into four bodies for respective focused refinement, yielding pol II/TFIIF at 3.0 and 3.5 Å resolution for forms 1 and 2, TFIIE, TBP and TFIIB at 6.1 and 4.7 Å resolution for forms 1 and 2, Rad3, Tfb1, Ssl1 and Tfb4 at 3.9 and 4.6 Å resolution, and Ssl2, Tfb2 and Tfb5 at 7.4 and 7.44 Å resolution. All the reported resolutions are based on the gold-standard Fourier shell correlation using 0.143 criterion (32).

### Model building and refinement

For core PIC, the previous model from the yeast PIC (PDB: 7ML0) was used as an initial template. For TFIIH, the previous model from the 3.9 Å resolution cryo-EM structure in the form of DNA repair (PDB: 7K01) (33) was used as an initial model. Promoter DNA was manually built by combining short (∼10 bp) B-form DNA segments. A combined model containing core PIC, TFIIH and promoter DNA was iteratively subjected to manual refinement (a combination of real-space refinement, regularization and rigid body fit of domains) with Coot (34) and rigid body refinement with Phenix 1.16 (35). Through the refinement, secondary structures and base pairs of DNA double helix were maintained. The structural refinement statistics are summarized in Supplementary Table S1. All figures were generated using UCSF Chimera (36).

## Results

### The hydrophobic loop of Tfb3 is a prerequisite for distal TSS utilization

**Figure 2.**
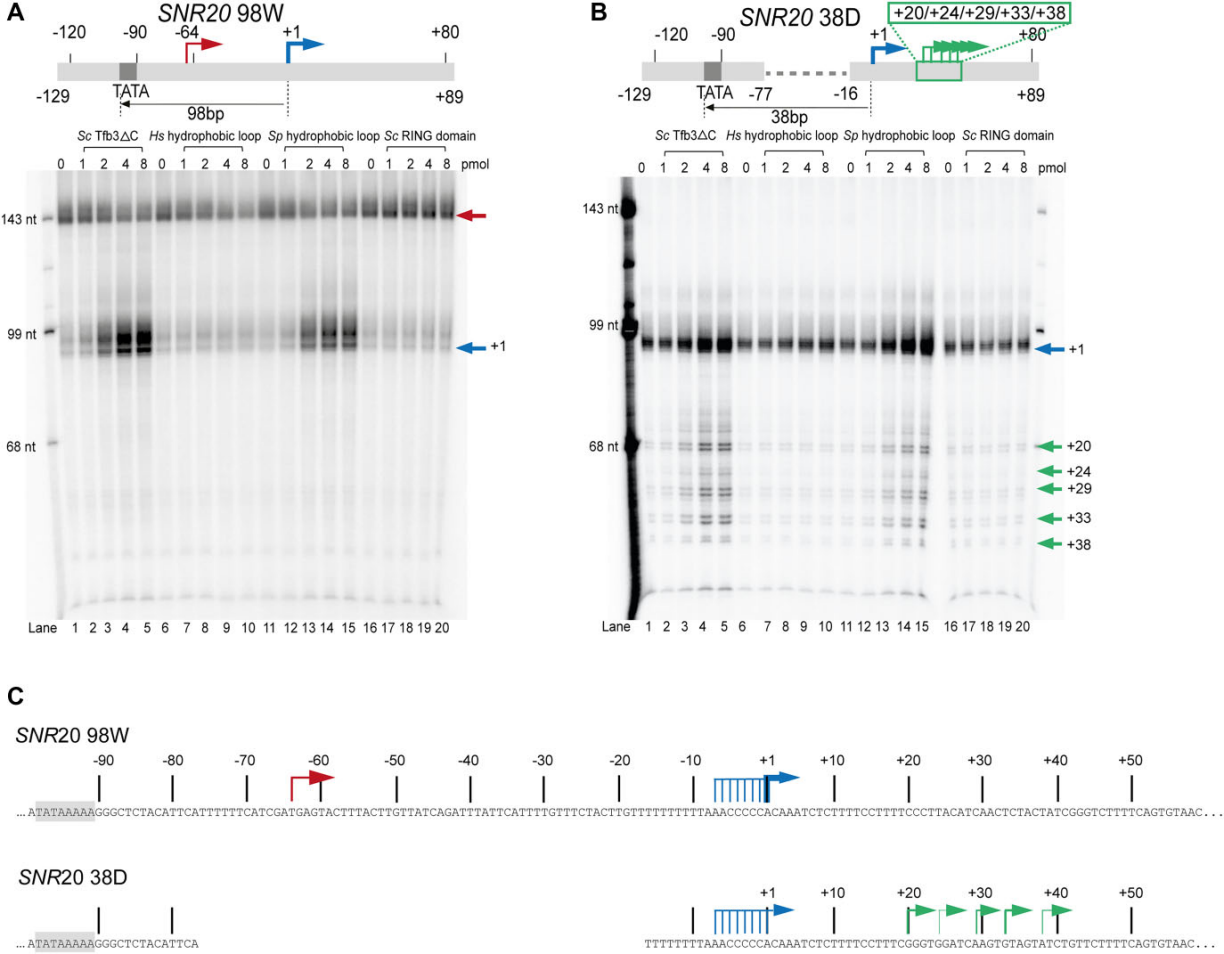
Run-off transcription assay with Tfb3ΔC hydrophobic loop (HL) mutants. (**A**) Run-off transcription with increasing amounts of WT *Sc*Tfb3ΔC (lanes 1–5), Tfb3ΔC with *Hs*HL (lanes 6–10), Tfb3ΔC with *Sp*HL (lanes 11–15) and *Sc*RING domain (lanes 16–20). 1.3 pmol of *SNR20* 98W promoter DNA fragment (–129/+89) was combined with TFIIA (2 pmol), TFIIB (6 pmol), TBP (12 pmol), Sub1 (2 pmol), TFIIE (3 pmol), TFIIF (5.2 pmol), core TFIIH (2 pmol), pol II (5.2 pmol) and Tfb3ΔC or its variants (amounts indicated above each lane). Transcripts initiated from upstream and downstream TSSs are indicated by red and blue arrows, respectively. (**B**) Same as panel (A) with *SNR20* 38D promoter DNA fragment (–129/+89). (**C**) A schematic of *SNR20* 98W (top) and *SNR20* 38D (bottom) promoters. TSSs were validated by primer extension analysis (Supplementary Figure S3). TSSs at −7 to −1 may result from slippage synthesis initiated at +1.

Using *S. cerevisiae* as a model system, we set up an *in vitro* reconstituted system containing pol II, GTFs including the seven-subunit core TFIIH (excluding holoTFIIH) and an *SNR20* promoter fragment (*SNR20* 98W) as an initiation template (25). In this promoter, a minor TSS at −4 on our previous *SNR20* 91W promoters (25) was disrupted by mutating two base pairs at positions −5 and −4 from TG to CC, thus carrying only a single A_−8_Y_−1_R_+1_ motif (Y = C or T, R = A or G) as a strong TSS at +1. This TFIIK-independent transcription system supported initiation almost exclusively ∼34 bp downstream of the TATA box. The position of this upstream TSS was validated by primer extension (see below; Supplementary Figure S3). Addition of TFIIK or recombinant Tfb3ΔC (residues 1–148) restored initiation from downstream TSSs (Supplementary Figure S2), whereas addition of the RING domain (residues 1–70) failed to do so (Figure 2A), suggesting that Tfb3ΔC is a prerequisite for distal TSS utilization presumably by bridging between pol II and TFIIH through the RING domain (residues 1–70) and the Rad3-binding domain (residues 97–140) (Figure 1A and B). No transcription was obtained from either upstream or downstream TSSs when the core TFIIH was omitted (Supplementary Figure S2), confirming the essential role of core TFIIH in the reaction. By accounting for the number of U residues labeled in transcripts, the transcription level from the downstream TSS relative to the upstream TSS was 8.5 ± 3.7-fold (*n* = 2) in the presence of Tfb3ΔC compared to 1.1 ± 0.4-fold in the absence of Tfb3ΔC (lane 5 versus lane 1 in Figure 2A). Tfb3ΔC evidently enhanced TSS scanning activity; however, whether Tfb3ΔC might also enhance overall PIC activity remains to be determined.

Leveraging the experimental advantages of the recombinant form of Tfb3ΔC in this system, we investigated a requirement for the Tfb3 hydrophobic loop (HL)(Figure 1A) for TSS scanning. Two Tfb3ΔC mutants were generated by swapping eight residues of the *Sc*HL (Ile46, Phe47, Ser48, Leu49, Gly50, Pro51, Ala52 and Gln53) of recombinant Tfb3ΔC (residues 1–148) for those in humans (Tfb3ΔC-*Hs*HL) or *S. pombe* (Tfb3ΔC-*Sp*HL). These Tfb3ΔC mutants were highly purified and eluted similarly to Tfb3ΔC by size exclusion chromatography (Supplementary Figure S1B). Addition of the chimeric Tfb3ΔC-*Sp*HL to the TFIIK-independent system supported initiation from downstream TSSs, although to a lesser extent than Tfb3ΔC (lanes 1–5 versus lanes 11–15 in Figure 2A). In contrast, the chimeric Tfb3ΔC-*Hs*HL had no effect on transcription (lanes 6–10 in Figure 2A).

To further support the requirement for the Tfb3 hydrophobic loop for TSS scanning, we created a promoter variant by bringing the TSS of the *SNR20* promoter from the wild-type location 98 bp downstream of the TATA box to location 38 bp downstream (*SNR20* 38D) (Figure 2B). Addition of Tfb3ΔC into the TFIIK-independent system with the *SNR20* 38D promoter supported transcription initiation from the +1 TSS (indicated in blue arrow) and, to a lesser extent, from TSSs located at positions +20 to +38 (lanes 1–5 in Figure 2B) corresponding to TSS scanning ∼58–76 bp downstream of the TATA box. Tfb3ΔC-*Sp*HL exhibited similar TSS scanning activity but to a lesser extent than Tfb3ΔC (lanes 11–15 in Figure 2B). In contrast, the chimeric Tfb3ΔC-*Hs*HL had no effect on transcription (lanes 6–10 in Figure 2B). These results strongly indicate a critical role of the hydrophobic loop of Tfb3 but not MAT1 in distal TSS utilization.

To understand how TSSs are preferentially selected among all possible TSSs on the two promoters, we performed primer extension to determine accurate positions of TSSs though not for quantitative analysis (Figure 2C and Supplementary Figure S3). We found that the strongest TSS at +1 originated from A_−8_Y_−1_R_+1_, while other less efficient TSSs originated from Y_−1_R_+1_ initiator elements, except TSSs at −7 to −1, where transcripts might actually initiate at +1 but extend at their 5′ end through slippage (37,38). In contrast, the upstream TSS of *SNR20* 98W located ∼34 bp downstream of the TATA box originated from a non-Y_−1_R_+1_ sequence. Thus, initiator sequences and distance may be controlling the responsiveness of upstream TSS usage to Tfb3ΔC *in vitro*. *In vivo*, TSS sequence controls initiation efficiency over a wide range (12–14,17).

### Tfb3 Pro51 is required for distal TSS utilization and conserved in fungi, but absent in metazoans

**Figure 3.**
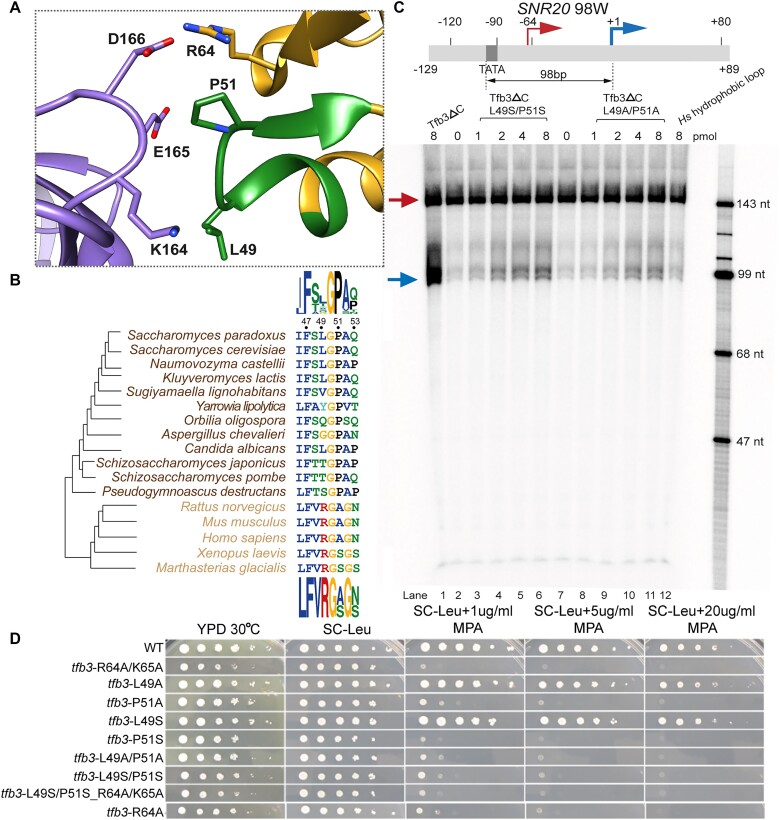
Pro51 confers distal TSS utilization in *S. cerevisiae*. (**A**) Interface between the Tfb3 hydrophobic loop (green) and Rpb7 (purple). (**B**) Multiple sequence alignment of the hydrophobic loop between fungi (brown) and metazoans (orange). Sequence logos in fungi and metazoans are displayed at the top and the bottom, respectively. (**C**) Run-off transcription with Tfb3ΔC (lane 1), Tfb3ΔC-L49S/P51S (lanes 2–6), Tfb3ΔC-L49A/P51A (lanes 7–11) and Tfb3ΔC*-Hs*HL (lane 12). Amounts of Tfb3ΔC and mutants added into the reactions are indicated above each lane. The assay was performed with *SNR20* 98W promoter DNA (–129/+89) as in Figure 2A. Transcripts initiated from upstream and downstream TSSs are indicated by red and blue arrows. (**D**) Growth phenotypes of *tfb3* mutants. Ten-fold serial dilutions of saturated cultures of *TFB3* WT and mutant strains plated on different media at 30°C. YPD is rich medium with dextrose as a carbon source. SC-Leu is defined as complete medium lacking leucine. MPA was added to this medium (SC-Leu + MPA) to 1, 5 and 20 μg/ml final concentrations, showing that P51A and P51S are sensitive to this drug, indicative of defects in TSS scanning at the *IMD2* gene, which is required for resistance. Pictures used are of day 2 for YPD, day 3 for SC-Leu and day 4 for the plates with varying concentrations of MPA.

To pinpoint residue(s) of the Tfb3 hydrophobic loop responsible for TSS scanning, we mutated Leu49 and Pro51, both of which are in contact with long aliphatic side chains of Lys164 and Glu165 of Rpb7 in yeast PICs (Figure 3A). Double mutants (L49S/P51S and L49A/P51A) of Tfb3ΔC greatly diminished transcription initiation from the downstream TSSs compared to Tfb3ΔC (Figure 3C), suggesting a critical role of Pro51 and/or Leu49 of Tfb3 in TSS scanning in yeast. It may be noted that the scanning activity of the double mutants was slightly higher than the chimeric Tfb3ΔC-*Hs*HL (compare with lane 12 in Figure 3C). The other six residues of the Tfb3 hydrophobic loop other than Leu49 and Pro51 may also contribute to TSS scanning *in vitro*.

To further support the role of Pro51 and/or Leu49 in TSS scanning, we tested these mutations *in vivo* by monitoring sensitivity to the drug MPA that correlates with the inability to use a downstream TSS at the *IMD2* gene (10,12). We found that *tfb3* alleles P51A or P51S, but not L49A or L49S, were as sensitive to MPA as our previous *tfb3* alleles R64A/K65A (26) (Figure 3D), suggesting a contribution of Pro51 to TSS scanning *in vivo*. Our mutational analysis aligns well with the observation that Pro51, rather than Leu49, exhibits extremely high conservation (∼100%) among fungi (Figure 3B), and that Pro51 is exclusively substituted by alanine or serine in metazoans. Altogether, Tfb3 Pro51 is likely to be a key determinant for downstream TSS utilization in yeast. Notably, the mutant L49S/P51S_R64A/K65A exhibited a similar MPA sensitivity and growth to either L49S/P51S or R64A/K65A alone (Figure 3D), further supporting that the hydrophobic and electrostatic interactions both contribute to the same function for downstream TSS utilization by stabilizing the Rpb7–Tfb3 interface. Also, it is important to note that these TSS mutants retain some degree of TSS scanning activity *in vivo* (see the ‘Discussion’ section).

### TFIIH in the PIC lacking TFIIK varies in orientation but maintains DNA engagement

**Figure 4.**
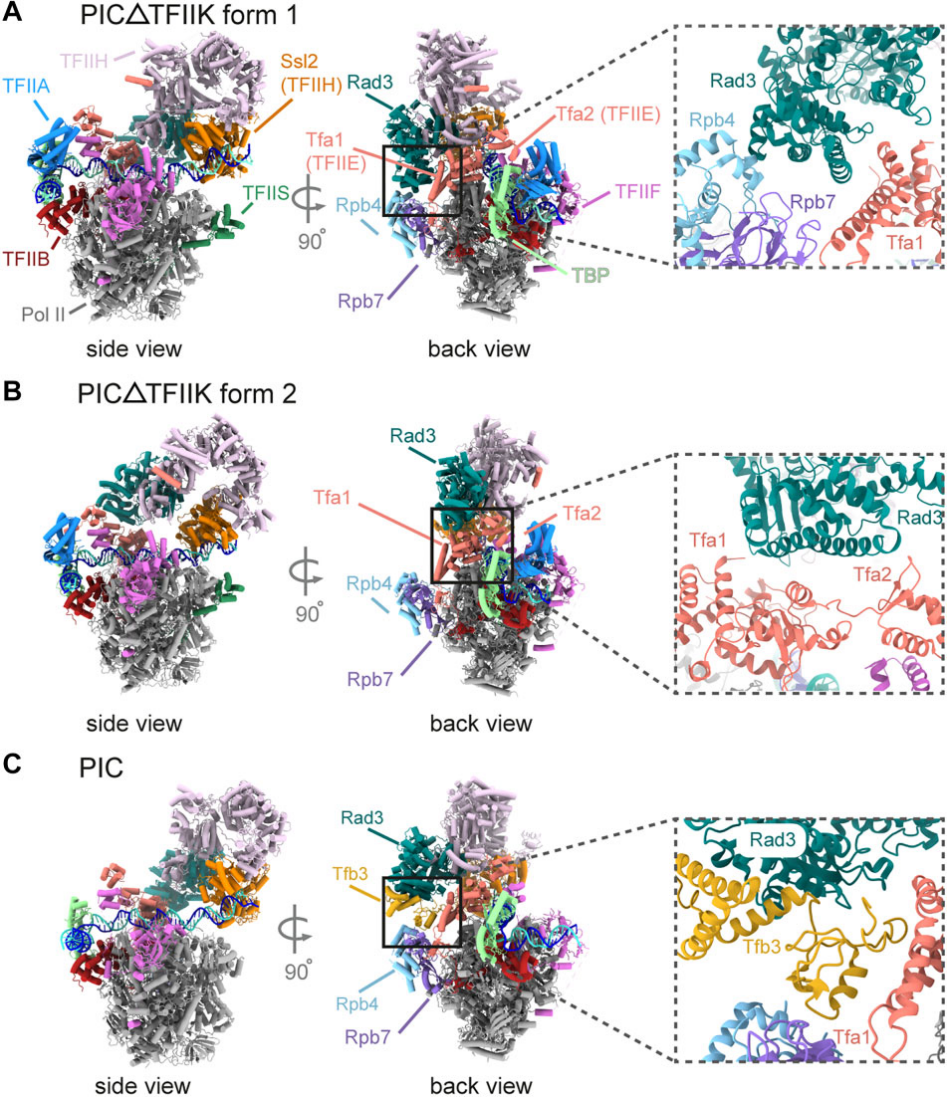
Cryo-EM structures of PIC-ΔTFIIK. (**A**) Cryo-EM map of form 1 of PIC-ΔTFIIK in side (left) and back (right) views with a corresponding model in the lower panel. Rad3 (sea green) is in direct contact with Rpb4 (light blue) and Tfa1 (salmon). (**B**) Same as panel (A), but for form 2. (**C**) Structure of the PIC containing TFIIK (PDB ID: 7ML0) is shown as cartoon in the same orientations as in panels (A) and (B).

Mutations of the Tfb3 hydrophobic loop or omission of TFIIK failed to initiate transcription from downstream TSSs, but not the upstream TSS, a characteristic of metazoan transcription. To determine the physical basis of this altered biochemical activity, a cryo-EM structure of yeast PIC lacking TFIIK was determined in the nucleotide-free state (Figure 4). 3D classification revealed two distinct forms, forms 1 and 2, accounting for 138 691 and 90 136 particles, at 3.7 and 3.8 Å resolution, respectively (Figure 4A and B, and Supplementary Figure S4). Both forms showed little difference in the locations of GTFs, compared to previous structures of PICs containing TFIIK (compare with Figure 4C). Due to the absence of TFIIK (Tfb3) that mediates interaction between TFIIH (Rad3) and Pol II (Rpb7), the ARCH domain of Rad3 is in direct contact with Rpb4 and Tfa1 (TFIIE) in form 1, and with the Tfa2 WH (TFIIE) domain in form 2 (insets in Figure 4A and B). Consequently, two forms differ in the orientation of TFIIH (Figure 4 and Supplementary Figure S5) as well as the path of downstream DNA (Supplementary Figure S6) compared to the PIC with TFIIK. This considerable variability in the orientation of TFIIH likely impairs downstream TSS scanning after promoter opening. Notably, despite such considerable variability in the orientation of TFIIH and DNA path, Ssl2 engages DNA, ∼47 bp downstream the TATA box, similarly to the PIC with TFIIK (Supplementary Figure S5A). An ∼13-bp segment of DNA double helix is bent, deep within the DNA-binding groove between the two ATPase domains of Ssl2 as in the canonical pre-translocation state [also referred to as a strong-binding state (26)] (Supplementary Figure S5C and D). These observations underscore the requirement for core TFIIH including Ssl2 for promoter opening and initiation from the upstream TSS even in the PIC lacking TFIIK (Supplementary Figure S2A).

## Discussion

One long-standing question has been how metazoan PICs choose to start at the upstream location whereas yeast PICs alternatively begin scanning and use a downstream location. Our biochemical, genetic and structural evidence suggests that downstream TSS utilization is at least partially attributable to a gain-of-function mutation in the hydrophobic loop of Tfb3, acquired during fungal evolution. Specifically, Pro51 is identified as a prerequisite for downstream TSS utilization. When Pro51 is mutated to alanine or serine as observed in metazoans (Figure 3), TSS utilization *in vitro* becomes virtually restricted to the upstream site. Moreover, *S. cerevisiae* Tfb3ΔC containing the hydrophobic loop from *S. pombe* facilitates TSS scanning to a lesser extent than *S. cerevisiae* Tfb3ΔC (Figure 2A), consistent with the different extent in TSS scanning *in vitro* between *S. cerevisiae* and *S. pombe* (20,21,39). It should be noted that these TSS mutations retain some degree of TSS scanning activity *in vivo*. This different sensitivity between *in vivo* and *in vitro* where *in vivo* scanning is more robust than *in vitro* remains to be determined. Additional factors or functions in the PIC may redundantly enforce downstream TSS usage *in vivo*. Additionally, promoter sequences have also co-evolved with the scanning mechanism, such as A richness upstream of the TSS and the specific enrichment of −8A in *Saccharomyces* acquired after the split with *Yarrowia lipolytica* during evolution (13). As such, evolution acquiring downstream TSS scanning likely occurred through a stepwise process involving both PIC components and promoter sequences, as proposed in (13).

We have determined two forms of the cryo-EM structure of PIC-ΔTFIIK in the nucleotide-free state. Except for the pronounced variability of TFIIH, there is little difference in the general picture between PIC-ΔTFIIK and the canonical form of yeast PICs such as PIC1 (26) (Supplementary Figure S5): in both forms, the downstream DNA was bound to Ssl2, ∼47 bp downstream of the TATA; TFIIH adopted the pre-translocation state (i.e. strong-binding state), in which an ∼13-bp segment of DNA double helix is located deep within the DNA-binding groove between the two ATPase domains of Ssl2. We did not observe the other forms of PICs such as PIC2 or PIC3 (26), which differ from PIC1 in the location of TFIIH by repositioning of Ssl2 on DNA by one turn of double-stranded DNA (∼10 bp) and thus likely represent transient PICs, for example, after the bubble collapse between successive rounds of TSS scanning. Notably, there are differences in DNA path between two forms of PIC-ΔTFIIK (Supplementary Figure S6B): in form 1, the DNA is suspended above the pol II cleft, in contact only with GTFs and not with pol II, as in canonical yeast PICs, whereas in form 2, the DNA path at the downstream end of the PIC partially resides within the pol II cleft, and coincides with that in mammalian PICs. Whether this repositioning contributes to the lack of TSS scanning remains to be determined.

**Figure 5.**
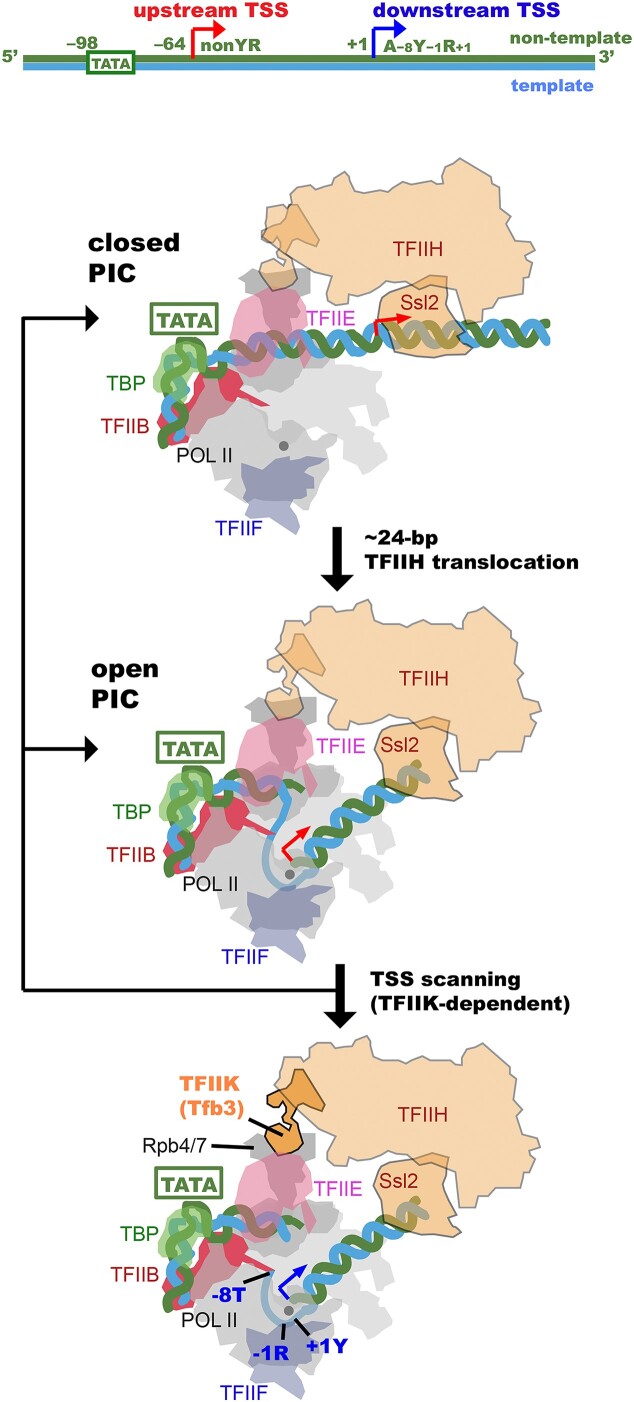
Schematic representation of TSS scanning in yeast. Schematic representation to show how the closed PIC (top panel) transitions to the open complex (middle panel) and facilitates TSS scanning (bottom panel) by the TFIIH translocation. DNA is blue and green. Tfb3 is orange. Upstream and downstream TSSs are indicated by red and blue arrows. The catalytic metal ion A in the pol II active center is indicated by a gray ball.

From a combination of much of available biochemical and structural information, we extended our previous mechanistic model of TSS scanning (18,25) (Figure 5). Previous single-molecule analysis of yeast PICs (18) revealed that Ssl2 bound to DNA, ∼47 bp downstream of the TATA box, draws 24 ± 2 bp into the pol II cleft, filling the single-stranded DNA-binding region of the active center (top and middle panels of Figure 5). This open complex represents a stable intermediate, as the TFIIH translocation was often paused after this initial 24-bp movement before further moving downstream for TSS scanning, and thus may allow for transcription initiation even without Y_−1_R_+1_ initiator elements. In the presence of TFIIK, further action of TFIIH displaces the single-stranded bubble by sliding it and forming a larger bubble accommodated by ‘scrunching’ DNA (18) for scanning downstream, through which a TSS can be selected among A_−8_Y_−1_R_+1_ or Y_−1_R_+1_ initiator elements with varying probabilities (bottom panel of Figure 5). If pol II cannot locate downstream TSS, the extended bubble opened by TFIIH collapses back to closed or open complexes, followed by repeated TSS scanning (18). We note that unanswered questions are what the mechanisms of non-scanning initiation by TFIIKΔ PICs are and what are the sequence determinants for TSS usage in upstream positions. We find that *SNR20* 98W upstream usage (a noncanonical non-Y_−1_R_+1_ initiator) is only partially suppressed by addition of Tfb3ΔC, while upstream usage for *SNR20* 38D (a canonical non-Y_−1_R_+1_ initiator on the edge of traditional scanning range) is promoted by the addition of Tfb3ΔC. These results suggest that there could be some complexity in sequence responsiveness best addressed by determining the sequence activity relationship for all sequences in the upstream region as has been done *in vivo* downstream TSS sequences at a synthetic *SNR37* promoter (14).

Our cryo-EM structure of PIC without TFIIK revealed that the Ssl2–DNA interaction is retained as in PICs with TFIIK, despite significant variability in the orientation of TFIIH. This interaction is in good agreement with our previous single-molecule analysis demonstrating the normal TFIIH processivity in PIC-ΔTFIIK with respect to translocation distance and velocity (18). Hence, we hypothesize that the failure in downstream TSS utilization in PIC-ΔTFIIK is attributed to uncoupling of TFIIH translocation from pol II scanning. TFIIK (Tfb3) may prevent such uncoupling by supporting core TFIIH (Ssl2) positioning and orienting relative to the pol II downstream cleft, ensuring the delivery of downstream DNA to the pol II channel.

Lastly, it may be noted that, although the distinct TSS utilization is conferred between yeast and metazoans, a recent study in the mammalian system has shown that the translocase activity of TFIIH persists until the PIC transitions into the elongation complex (40), similar to observations in yeast PICs (26,41). The mechanism of how TFIIH functions in promoter opening and subsequent events, including initiation and the transition to RNA chain elongation (42,43), appears to be largely conserved.

## Supplementary Material

gkae805_Supplemental_File

## Data Availability

The cryo-EM maps have been deposited at the Electron Microscope Data Bank (EMDB) under accession codes EMD-42438 (PIC-ΔTFIIK form 1) and EMD-42437 (PIC-ΔTFIIK form 2), and are publicly available as of the date of publication. The atomic coordinates have been deposited at the Protein Data Bank (PDB) under accession codes 8UOT (PIC-ΔTFIIK form 1) and 8UOQ (PIC-ΔTFIIK form 2), and are publicly available as of the date of publication.
