# Supplementary material for: Transcription start site scanning requires the fungi-specific hydrophobic loop of Tfb3
Source: Nucleic Acids Res. 2024 Sep 17;52(19):11602–11. doi: 10.1093/nar/gkae805 (PMC11514446; doi:10.1093/nar/gkae805)
Supplement: gkae805_Supplemental_File [file gkae805_supplemental_file.pdf]

Transcription start site scanning requires the fungi-specific hydrophobic loop of Tfb3

Chun Yang[1], Pratik Basnet [2], Samah Sharmin[1], Hui Shen[3] , Craig D. Kaplan[2], and Kenji Murakami[1*]

[1] Department of Biochemistry and Biophysics, Perelman School of Medicine, University of Pennsylvania, Philadelphia, Pennsylvania, 19104, U.S.A.

[2] Department of Biological Sciences, University of Pittsburgh, Pittsburgh, Pennsylvania, 15260, U.S.A.

[3] School of Life Science and Technology, China Pharmaceutical University, Nanjing, 210009, China.

Corresponding author: Kenji Murakami
Email: kenjim@pennmedicine.upenn.edu

SUPPLEMENTARY DATA

Supplementary Table S1

Supplementary Figures S1-S6

Supplementary Table S1. Cryo-EM data collection and refinement statistics

	PIC-∆TFIIK form 1	PIC-∆TFIIK form 2
**Data collection and processing**		
Magnification	81,000	81,000
Voltage (kV)	300	300
Electron exposure ($e^-/Å^2$)	50	50
Defocus range (μm)	-1.0 to -2.5	-1.0 to -2.5
Pixel size (Å)	0.54	0.54
Map resolution (Å)(FSC 0.143)	3.0[a]/6.1[b]/3.9[c]/7.4[d]	3.5[a]/4.7[b]/4.6[c]/7.44[d]
EMDB entry	EMD-42438	EMD-42437
Final particle	138,691	90,136
**Model Refinement**		
Model resolution (Å) (FSC 0.5)	3.7	4.9
PDB entry	8UOT	8UOQ
**Model composition**		
Non-hydrogen atoms	70,523	70,481
Protein residues	8,540	8,540
Nucleotides	128	126
Ligands	18	18
**R.m.s deviations**		
Bond lengths (Å)	0.01	0.006
Bond angles (°)	1.091	1.036
MolProbity score	2.11	2.10
Clash score	12.00	11.65
Poor rotamer (%)	0.03	0.03
**Ramachandran plot**		
Favored (%)	90.98	91.13
Allowed (%)	8.95	8.83
Disallowed (%)	0.07	0.04
**Model vs. Data**		
CC (mask)	0.69	0.69
CC (box)	0.85	0.85
CC (volume)	0.69	0.70
CC (peaks)	0.64	0.62
CC (main chain)	0.76	0.76
CC (side chain)	0.77	0.77

[a]Pol II, TFIIF; [b]DNA, TBP, TFIIB, TFIIE; [c]TFIIH core; [d]Ssl2,Tfb5 and Tfb2;

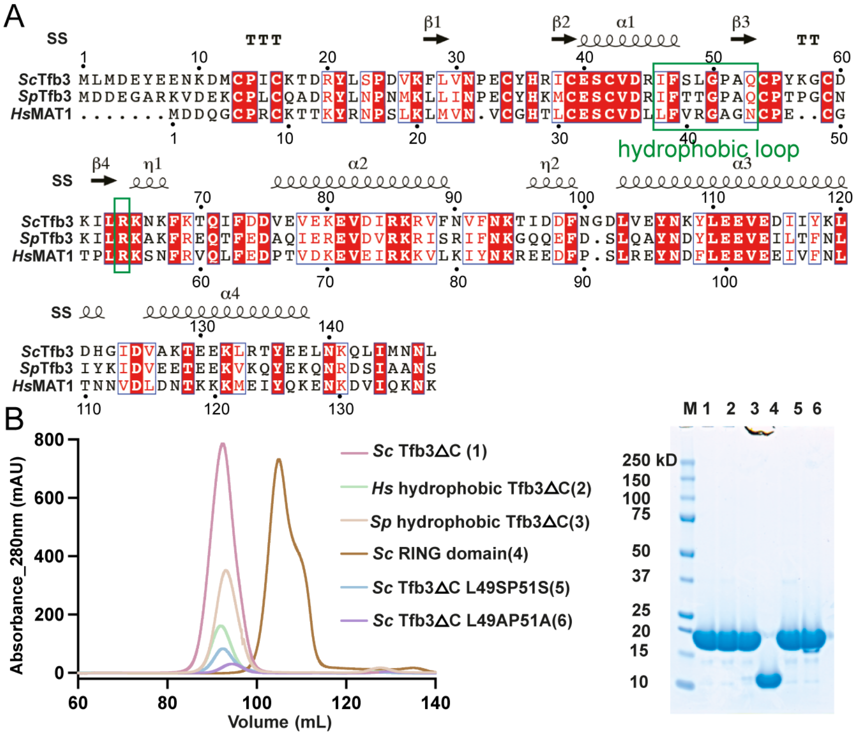

Supplementary Figure S1. Sequence alignment of Tfb3/MAT1. (A) Sequence alignment of Tfb3ΔC (residues 1-148) from *Saccharomyces cerevisiae* (*Sc*), *Schizosaccharomyces pombe* (*Sp*), and *Homo sapiens* (*Hs*) with secondary structure annotations from *Sc* PIC. The hydrophobic loop as well as Arg64 is indicated by green boxes. **(B)** Size exclusion chromatogram of purified Tfb3ΔC and HL mutants (left), and SDS-PAGE followed by Coomassie blue staining (right).

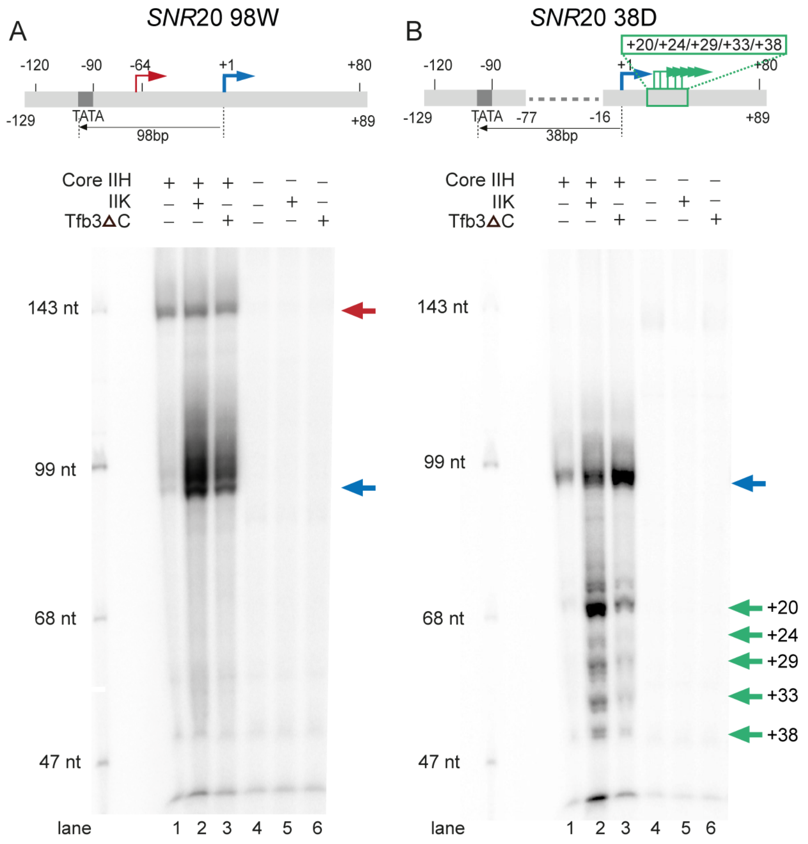

Supplementary Figure S2. Run-off transcription *in vitro* with *SNR20* 98W or *SNR20* 38D. The reactions of TFIIK-independent transcription involving 2 pmol of core TFIIH were performed with *SNR20* 98W **(A)** or *SNR20* 38D **(B)** as in Figure 2, with additional factors (8 pmol TFIIK, 8 pmol Tfb3ΔC) as indicated over the lanes.

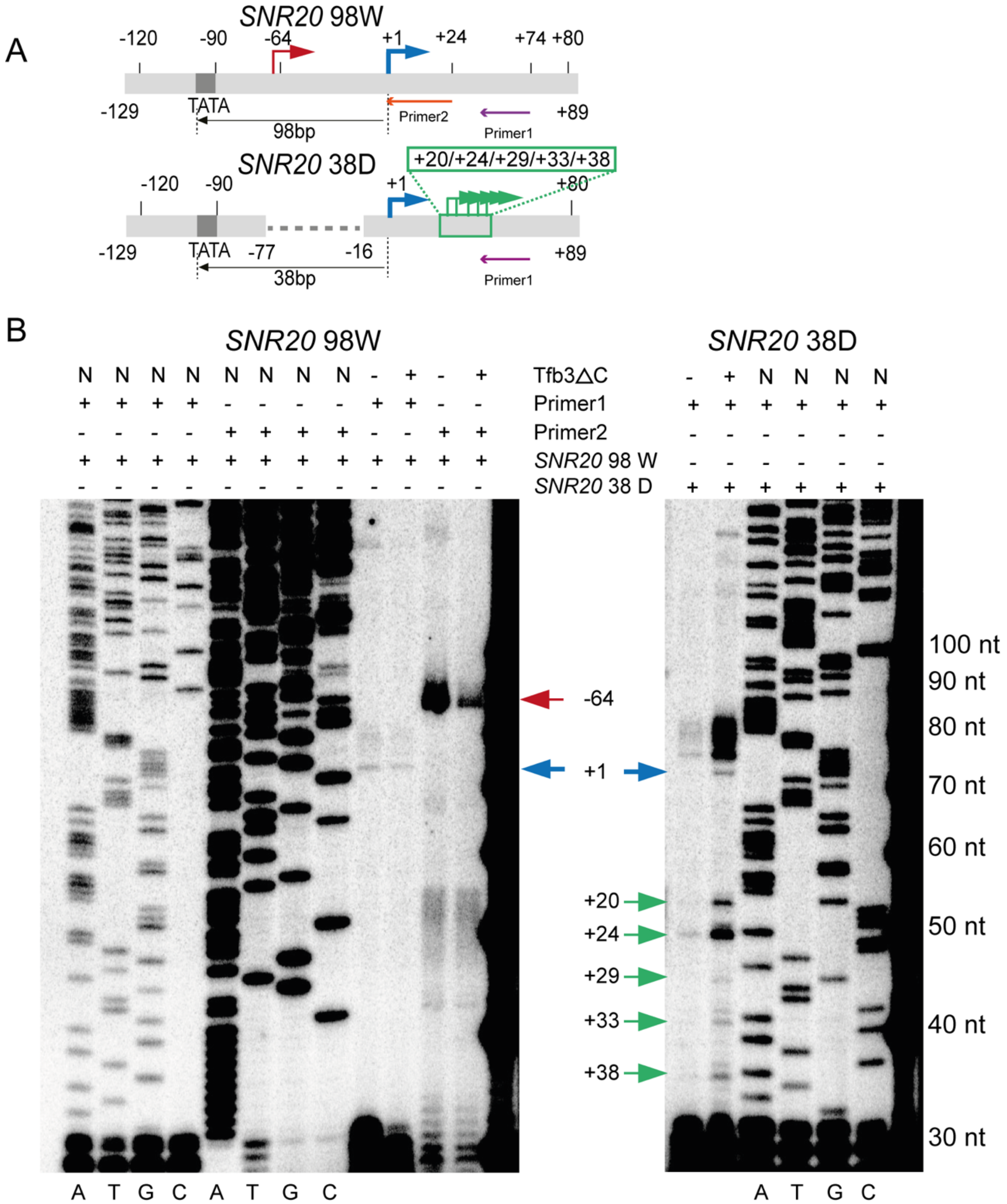

Supplementary Figrue S3. Primer extension analysis *in vitro* with *SNR20* 98W or *SNR20* 38D. Reactions were performed as in Figure S2 with or without Tfb3ΔC as indicated over lanes, followed by primer extension analysis (see Methods).

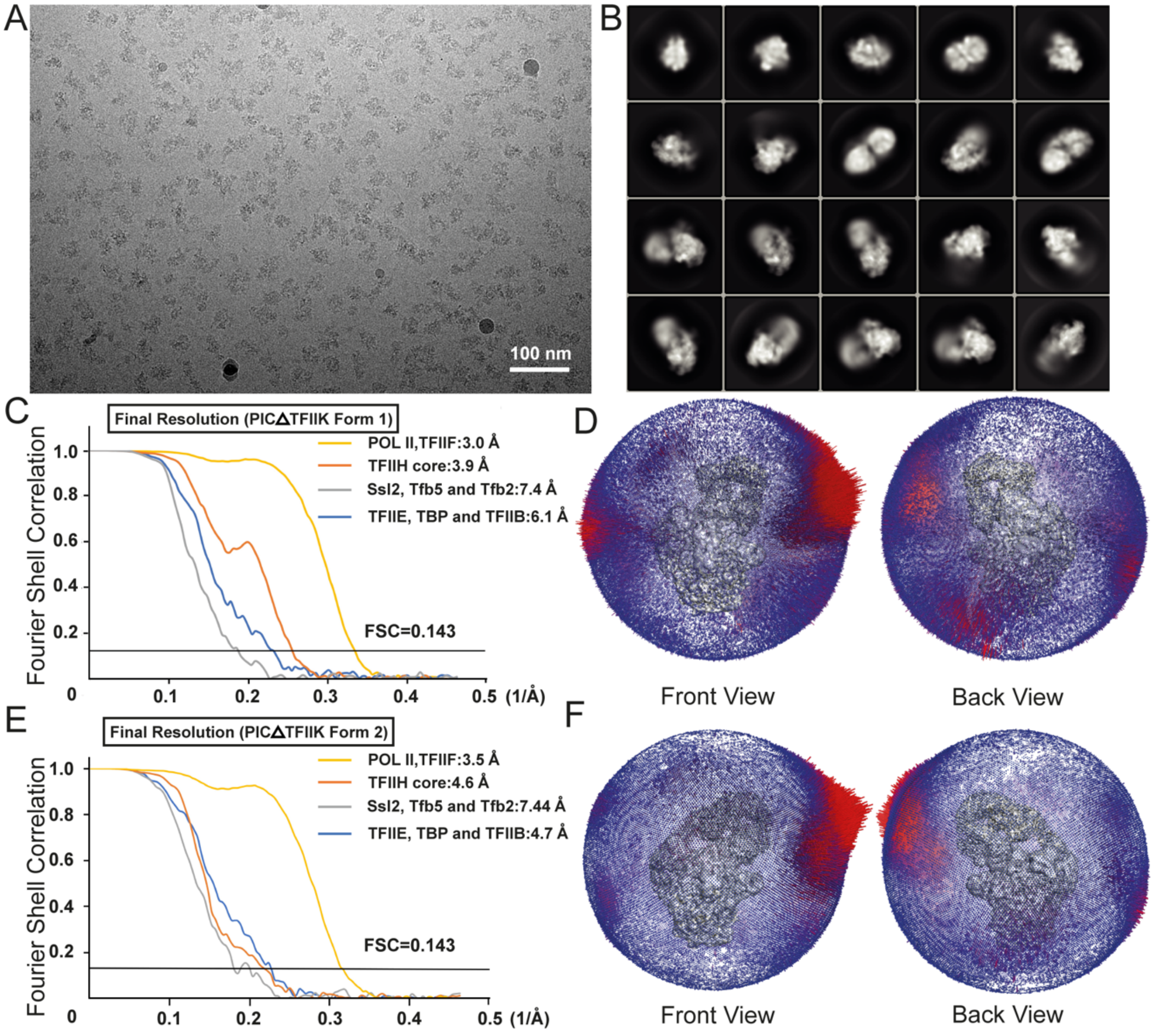

A
100 nm
B
C
Final Resolution (PICΔTFIIK Form 1)
POL II,TFIIF:3.0 Å
TFIIH core:3.9 Å
Ssl2, Tfb5 and Tfb2:7.4 Å
TFIIE, TBP and TFIIB:6.1 Å
FSC=0.143
Fourier Shell Correlation
1.0
0.8
0.6
0.4
0.2
0
0.1
0.2
0.3
0.4
0.5 (1/Å)
D
Front View
Back View
E
Final Resolution (PICΔTFIIK Form 2)
POL II,TFIIF:3.5 Å
TFIIH core:4.6 Å
Ssl2, Tfb5 and Tfb2:7.44 Å
TFIIE, TBP and TFIIB:4.7 Å
FSC=0.143
Fourier Shell Correlation
F
Front View
Back View

G

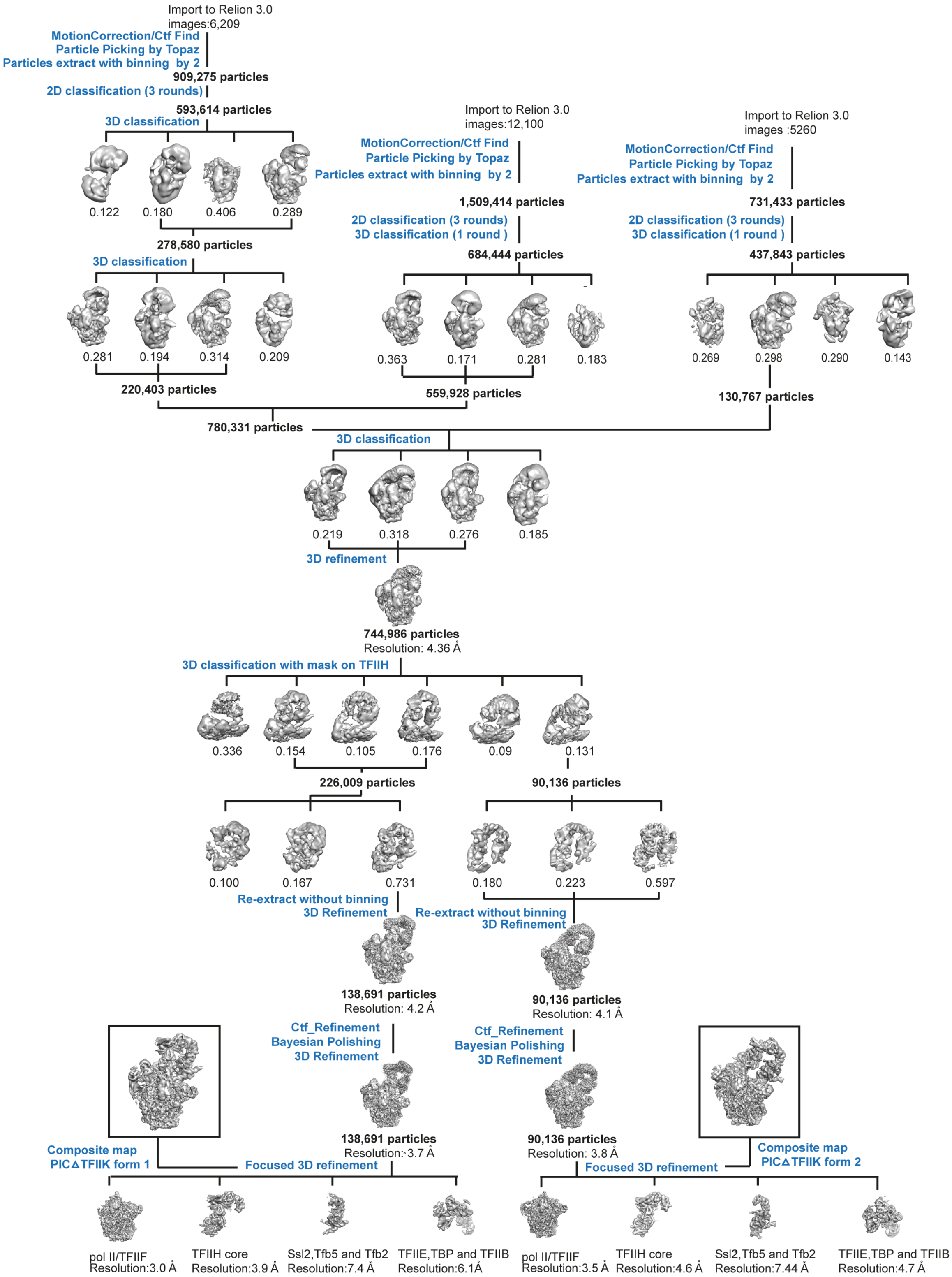

Import to Relion 3.0
images:6,209
MotionCorrection/Ctf Find
Particle Picking by Topaz
Particles extract with binning by 2
909,275 particles
2D classification (3 rounds)
593,614 particles
3D classification
0.122
0.180
0.406
0.289
278,580 particles
3D classification
0.281
0.194
0.314
0.209
220,403 particles
Import to Relion 3.0
images:12,100
MotionCorrection/Ctf Find
Particle Picking by Topaz
Particles extract with binning by 2
1,509,414 particles
2D classification (3 rounds)
3D classification (1 round)
684,444 particles
0.363
0.171
0.281
0.183
559,928 particles
Import to Relion 3.0
images :5260
MotionCorrection/Ctf Find
Particle Picking by Topaz
Particles extract with binning by 2
731,433 particles
2D classification (3 rounds)
3D classification (1 round)
437,843 particles
0.269
0.298
0.290
0.143
130,767 particles
780,331 particles
3D classification
0.219
0.318
0.276
0.185
3D refinement
744,986 particles
Resolution: 4.36 Å
3D classification with mask on TFIIH
0.336
0.154
0.105
0.176
0.09
0.131
226,009 particles
90,136 particles
0.100
0.167
0.731
0.180
0.223
0.597
Re-extract without binning
3D Refinement
Re-extract without binning
3D Refinement
138,691 particles
Resolution: 4.2 Å
90,136 particles
Resolution: 4.1 Å
Ctf_Refinement
Bayesian Polishing
3D Refinement
Ctf_Refinement
Bayesian Polishing
3D Refinement
138,691 particles
Resolution: 3.7 Å
90,136 particles
Resolution: 3.8 Å
Composite map
PICΔTFIIK form 1
Focused 3D refinement
Focused 3D refinement
Composite map
PICΔTFIIK form 2
pol II/TFIIF
Resolution:3.0 Å
TFIIH core
Resolution:3.9 Å
Ssl2,Tfb5 and Tfb2
Resolution:7.4 Å
TFIIE,TBP and TFIIB
Resolution:6.1Å
pol II/TFIIF
Resolution:3.5 Å
TFIIH core
Resolution:4.6 Å
Ssl2,Tfb5 and Tfb2
Resolution:7.44 Å
TFIIE,TBP and TFIIB
Resolution:4.7 Å

Supplementary Figure S4. Cryo-EM data analysis of PIC-ΔTFIIK. (A) A representative raw micrograph of PIC-ΔTFIIK. **(B)** Representative 2D class averages of PIC-ΔTFIIK. **(C-D)** FSC curves (C) and corresponding angular distribution (D) of particles of PICΔTFIIK form 1 from front view (left) and back view (right). **(E-F)** Same as (C-D) but for PIC-ΔTFIIK form 2. **(G)** Cryo-EM processing pipeline of PIC-ΔTFIIK.

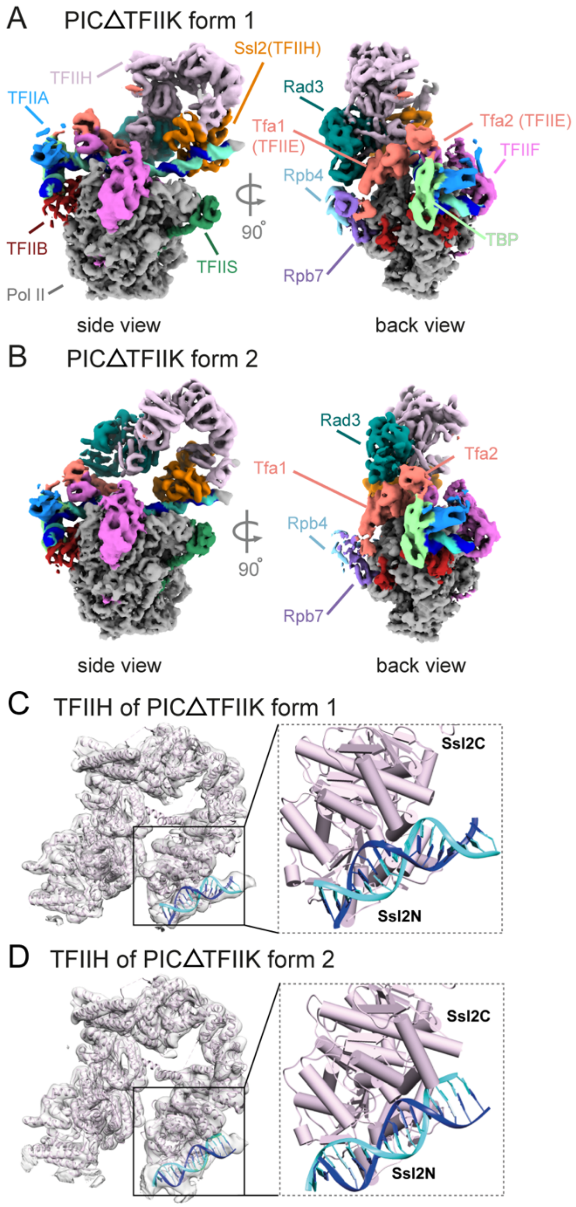

Supplementary Figure S5. Cryo-EM maps of PIC-ΔTFIIK
(A) Cryo-EM map of form 1 of PIC-ΔTFIIK in side (left) and back (right) views. The map corresponds to the model in Figure 4A. **(B)** Same as (A) but for form 2. **(C-D)** Composite density maps and models for TFIIH in the strong binding state, corresponding to maps in (A) and (B).

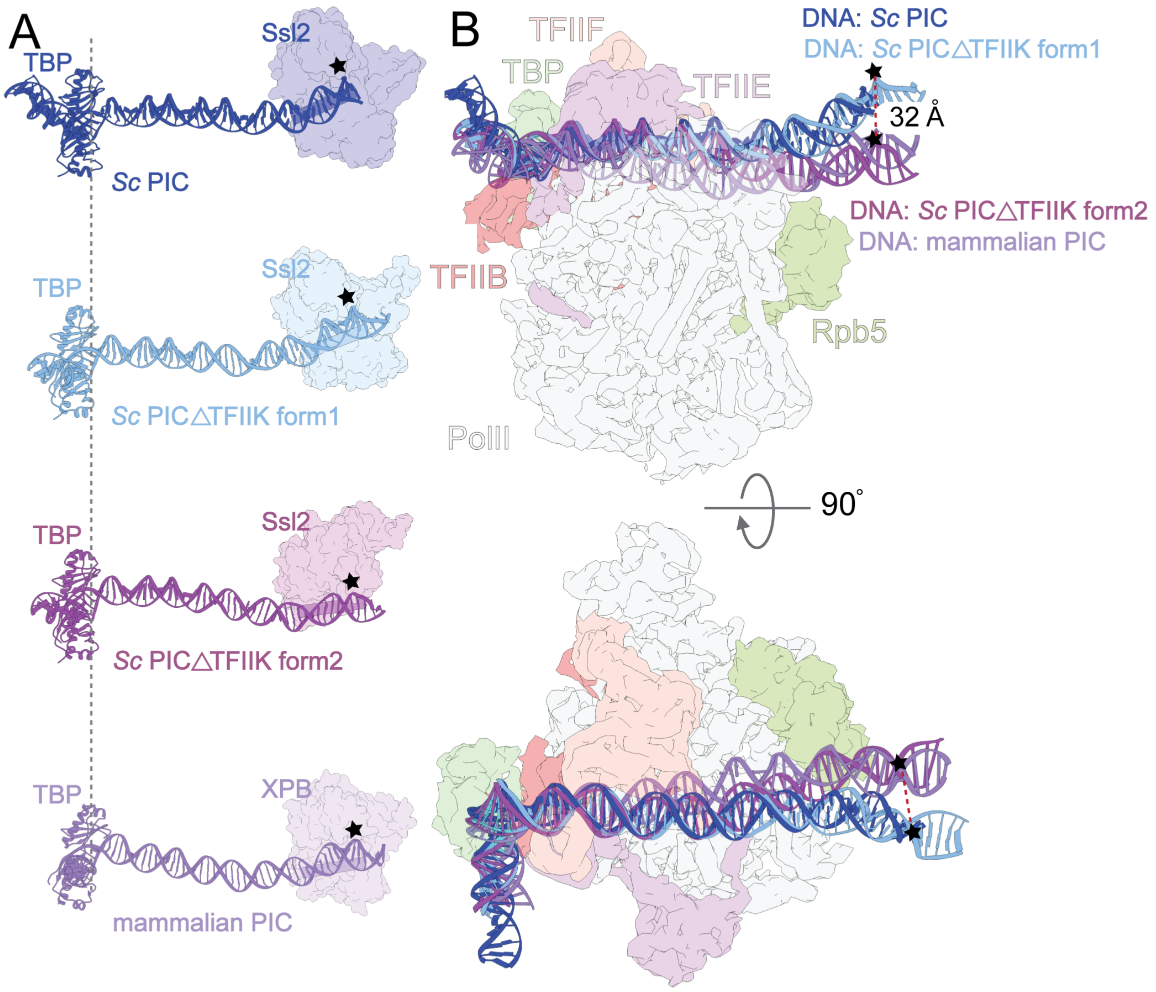

Supplementary Figure S6. Comparison of paths of promoter DNA in *Sc* PIC, mammalian PIC and *Sc* PIC-ΔIIK (forms 1 and 2)

(A) Relationship between promoter DNA, TBP, and Ssl2/XPB. Ssl2/XPB bind DNA 47 bp downstream of the TATA box (indicated by a star) in all forms. Structures are aligned at the first T of the TATA box (dashed line). **(B)** Superposition of *Sc* PIC (PDB ID: 7ML0), *Sc* PIC- delta IIK form1, *Sc* PIC- delta IIK form 2 and mammalian PIC (PDB ID: 7NVZ) from side view (top) and top view (bottom). TFIIH is omitted for clarity.
